# Two new species of *Hiptage* (Malpighiaceae) from Yunnan, Southwest of China

**DOI:** 10.3897/phytokeys.110.28673

**Published:** 2018-11-05

**Authors:** Bin Yang, Hong-Bo Ding, Jian-Wu Li, Yun-Hong Tan

**Affiliations:** 1 Southeast Asia Biodiversity Research Institute, Chinese Academy of Sciences, Yezin, Nay Pyi Taw 05282, Myanmar Southeast Asia Biodiversity Research Institute, Chinese Academy of Sciences Yezin China; 2 Centre for Integrative Conservation, Xishuangbanna Tropical Botanical Garden, Chinese Academy of Sciences, Menglun, Mengla, Yunnan 666303, PR China Xishuangbanna Tropical Botanical Garden, Chinese Academy of Sciences Yunnan China

**Keywords:** *
Hiptage
*, Malpighiaceae, samara, Yunnan, China

## Abstract

*Hiptagepauciflora* Y.H. Tan & Bin Yang and *Hiptageferruginea* Y.H. Tan & Bin Yang, two new species of Malpighiaceae from Yunnan, South-western China are here described and illustrated. Morphologically, *H.pauciflora* Y.H. Tan & Bin Yang is similar to *H.benghalensis* (L.) Kurz and *H.multiflora* F.N. Wei; *H.ferruginea* Y.H. Tan & Bin Yang is similar to *H.calcicola* Sirirugsa. The major differences amongst these species are outlined and discussed. A diagnostic key to the two new species of *Hiptage* and their closely related species is provided.

## Introduction

*Hiptage* Gaertn. (Gaertner 1791) is one of the largest genera of Malpighiaceae with about 30 species of woody lianas and shrubs growing in forests of tropical South Asia, Indo-China Peninsula, Indonesia, Philippines and Southern China, including Hainan and Taiwan islands (Chen and Funston 2008, Ren et al. 2013). There are currently ten species of *Hiptage* recorded in Thailand (Sirirugsa 1987, 1991), ten species with seven endemic ones in China (Chen and Funston 2008) and 9 species in India (Srivastava 1992). So far, Ren (2015) recognised a total of 29 species in this genus.

The genus name, *Hiptage*, is derived from the Greek “hiptamai” which means to fly and refers to its distinctive three-winged fruit samaras, i.e. a larger middle wing and two smaller lateral wings, sometimes the dorsal wing is absent or present as a small crest, most species bear an elongated commissural gland on the calyx (Srivastava 1992, Anderson et al. 2006). *Hiptage* is also unique for having mirror-image flowers, with 1 long and 9 short unequal stamens (Ren et al. 2013, Qian et al. 2016). *H.benghalensis* (L.) Kurz is the most well-known species due to its attractive and fragrant flowers; it is often cultivated as a tropical ornamental plant in gardens (Anderson et al. 2006).

During floristic surveys in southern and south-western Yunnan from 2009 to 2017, some populations of *Hiptage* were discovered and some plants were cultivated in Xishuangbanna Tropical Botanical Garden (**XTBG**), Chinese Academy of Sciences (**CAS**). Based on detailed examination on the morphological and anatomical characters of the living plants and specimens and comparing them with the possible closely similar species, we found these populations representing two species distinct from the known 29 species so far (Sirirugsa 1991, Srivastava 1992, Chen and Funston 2008, Ren 2015). We draw a conclusion that those two species are new to science. Therefore, we described and illustrated the new species and compare them morphologically to its most similar congener.

## Material and methods

Measurements and assessments of morphological characters of the two possible new species *H.pauciflora* Y.H. Tan & Bin Yang and *H.ferruginea* Y.H. Tan & Bin Yang were based on dried specimens in herbarium and fresh materials in field observations and cultivation plants flowered and fruited in XTBG. They were compared with the morphologically similar species *H.benghalensis* (Sirirugsa 1991, Chen and Funston 2008), *H.multiflora* (Wei 1981, Chen and Funston 2008) and *H.calcicola* (Sirirugsa 1987, 1991) in affinities inferred from protologues, type specimens and cultivation plants in XTBG. Images of type specimens were gathered from JSTOR Global Plants (http://plants.jstor.org) and Chinese Virtual Herbarium (http://www.cvh.ac.cn). Conservation status evaluations of the new species were based on the International Union for Conservation of Nature guidelines (IUCN 2012).

## Taxonomy

### Hiptage pauciflora

Taxon classificationPlantaeMalpighialesMalpighiaceae

Y.H.Tan & Bin Yang sp. nov.

urn:lsid:ipni.org:names:77191587-1

Figure 1

#### Vernacular name.

Shao hua feng zheng guo (少花风筝果) (Chinese)

#### Diagnosis.

*Hiptagepauciflora* is similar to *H.benghalensis* in elliptic-oblong leaf blades, petal shape and size and also shares similarities with *H.multiflora* in having leaf marginal gland dots, sub-orbicular calyx glands and not decurrent to pedicel, obovate middle wing of samara, but differs from the former by lacking basal glands at the leaf base, calyx glands ovate or sub-orbicular to cordate, scarcely decurrent to pedicel, middle wing of the samara obovate-elliptic, lanceolate bracteoles 7–11 mm (vs. 1 mm); and differs from the latter by its fewer flowers, longer pedicels, without basal glands at the leaf base and elliptic sepals (vs. ovate).

#### Type.

CHINA. Yunnan Province, Menglian, Chengzi, limestone forest, the voucher from a cultivated plant at Xishuangbanna Tropical Botanical Garden, Chinese Academy of Sciences, 1 March 2017, *Y.H. Tan & B. Yang, XTBG-0013* (holotype, HITBC!).

#### Description.

Woody climbing shrubs. Young branches pubescent, older twigs with rounded lenticels, rough warts dot-like. Leaf blades ovate or ovate-elliptic to elliptic-oblong, 6.0–10.5 × 2.5–12.0 cm, base subcordate to cordate, apex attenuate to acuminate, lateral veins 5–8 pairs, without basal glands; young leaves silver-grey pubescent; mature leaves with upper surface glabrous, lower surface sparsely pubescent, conspicuous along the midvein, with 3–6 pairs marginal gland dots. Petiole 4–7 mm long, pubescent. Racemes axillary or terminal, 5–10 cm, silver-grey pubescent; peduncle 0.5–2 cm. Flowers pink; Bracts triangular to ovate-triangular, 1.5–2 mm, bracteoles lanceolate, 7–11 × 1–3 mm; Pedicels 1.8–2.9 cm long, pubescent, articulate at the middle to a little above the middle; Calyx with 1 gland, prominent, ovate or sub-orbicular to cordate, scarcely decurrent to pedicel; Sepals elliptic to ovate-elliptic, ca. 5–7 × 2.5–3 mm, apex obtuse, base truncate, pubescent outside; Petals 5 per flower, pink, with yellowish blotches, ovate-oblong to suborbicular, 1.2–1.4 × 0.8–1.1 cm, glabrous, base rounded to subcordate, apex rounded, margin fringed, claw 1–1.5 mm; Stamens 10, differing in size, the longest filaments 12–13 mm, circinate at apex, the short ones 5–8 mm, anther ca. 1.5–2 mm long. Ovary ca. 2.5–3 mm in diam., pilose; style1.2–1.4 cm long, glabrous, circinate at apex. Mericarps hairy, middle wing obovate-elliptic, puberulous. apex rounded, base obtuse, margin sometimes repand, 1.7–2.5 × 1–1.3 cm, lateral wings oblong, apex obtuse or rounded, margin sometimes crested, 1.3–1.6 × 0.3–0.5 cm; dorsal wing present or not, 2–4 × 1–2 mm.

**Figure 1.**
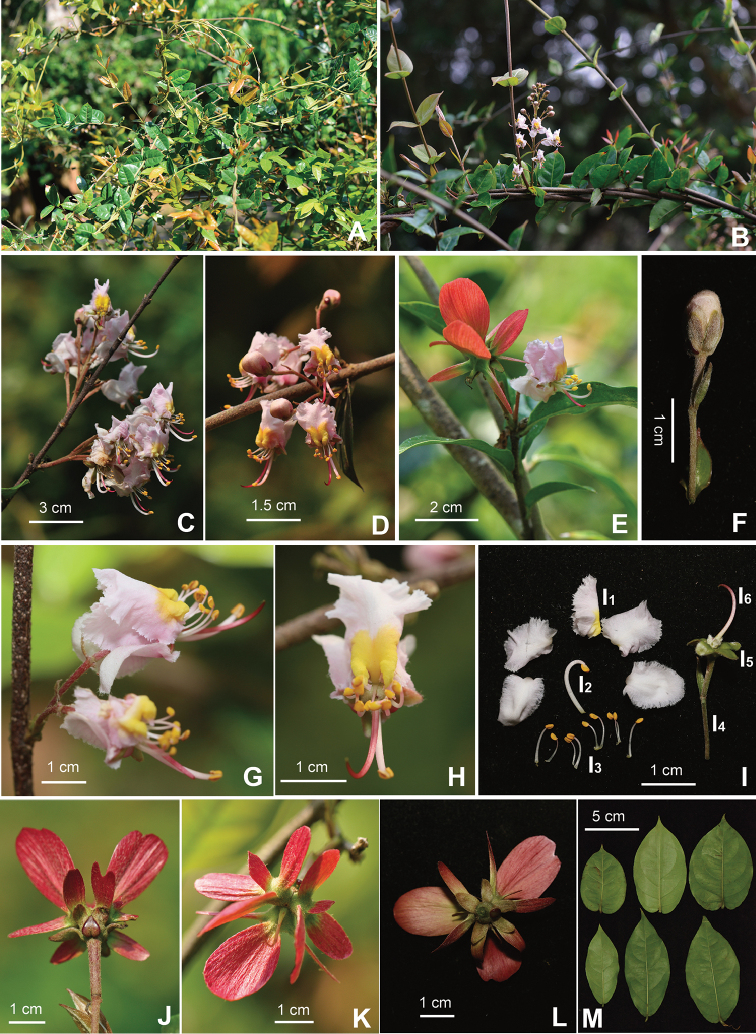
*Hiptagepauciflora* Y.H.Tan & Bin Yang, sp. nov. **A, B** Habit **C** Inflorescence (lateral view) **D** Inflorescence (frontal view) **E** Flowering branch **F** Flower bud **G** Flowers (lateral view) **H** Flower (frontal view) **I** Flower (I1 Petals; I2 the longest stamen; I3 the 9 short stamens; I4 Pedicel; I5 Calyx; I6 Style) **J** Samaras (lateral view and showing calyx gland) **K** Samaras (vertical view) **L** Samaras (dorsal view) **M** Leaves (adaxial view). Photographed by Y.H. Tan, H.B. Ding and B. Yang.

#### Phenology.

*Hiptagepauciflora* has been observed in flower at Xishuangbanna Tropical Botanic Garden under cultivation from the beginning of March and in fruit from March to April.

#### Etymology.

The species epithet refers to its inflorescence with fewer flowers, usually 1–8 flowers.

#### Distribution and habitat.

According to the introduction record, *H.pauciflora* was originally collected from Menglian, south-western Yunnan. Additional collections in the future may help to clarify its full distribution.

#### Conservation status.

Due to insufficient field surveys so far, very few details about its natural distribution and population status are currently known. The lack of sufficient data does not allow a final risk evaluation and the species might be regarded as data deficient (DD) according to the IUCN Red List Categories (IUCN 2012).

#### Specimen examined

(paratypes). CHINA. Yunnan, Menglian, Chengzi, limestone forest, 7 February 1976, *G. D. Tao 9082* (HITBC!, IBSC!). Menglian, from Mangxin to Chahe, 22°12.985'N, 99°35.292'E, alt. 987m, limestone forest, 29 March 2010, *E. D. Liu, W. Fang, W. Z. Ma & H. J. Dong 2376* (KUN!). Menglian, Chengzi, the voucher from a cultivated plant at Xishuangbanna Tropical Botanical Garden, Chinese Academy of Sciences, 8 March 2018, *B. Yang & H.B. Ding*, *XTBG 0044* (HITBC!).

### Hiptage ferruginea

Taxon classificationPlantaeMalpighialesMalpighiaceae

Y.H.Tan & Bin Yang sp. nov.

urn:lsid:ipni.org:names:77191588-1

Figure 2

#### Vernacular name.

Xiu mao feng zheng guo (锈毛风筝果) (Chinese)

#### Diagnosis.

*Hiptageferruginea* is similar to *H.calcicola* in elliptic leaf shape, hairy pedicels and calyx without glands, suborbicular petals; but differs in having marginal gland dots, without laminal gland dots (vs. without marginal gland dots, with laminal gland dots), pink petals (vs. white) larger size, claw 2.5–4 mm (vs. 1–2 mm), middle wings of samara obovate (vs. oblong).

#### Type.

CHINA. Yunnan, Mengla, Menglun, tropical seasonal moist limestone forest, 690 m a.s.l, *B. Yang & X.D. Zeng*, 4 March 2017, *XTBG0022* (holotype, HITBC!).

#### Description.

Scrambling shrubs. Young branches glabrescent, older twigs with rounded lenticels, rough, axillary buds sparsely pubescent. grey blades elliptic or elliptic-oblong, 6.0–7.5 × 2.2–2.8 cm, glabrous, surface without larminal gland dots, base cuneate to obtuse, with 1 pair of dark-coloured marginal glands near base, apex acute to attenuate, midrib prominent beneath, lateral veins 5–6 pairs. Petiole 5–7 mm long, glabrous. Racemes axillary or terminal, multiflorous, rust-coloured hairy, up to 6 cm. Flowers pink to pinkish-white; Bracts triangular to lanceolate, 1.5–2 mm, bracteoles ovate-triangular, ca.1 mm long; Pedicels 0.5–1.3 cm long, rust-coloured hairy, articulate below the middle; Calyx glands absent; Sepals elliptic to ovate-elliptic, ca. 2.5 × 2 mm, apex obtuse, base truncate, pubescent outside; Petals 5 per flower, spreading, suborbicular, 7–8 × 6–7 mm, pubescent outside, densely near base, glabrous inside, base rounded to truncate, apex rounded, margin fimbriate, claw 2.5–4 mm, adaxial pubescent; Stamens 10, differing in size, the longest filaments ca. 8–9 mm, the short ones 5–6 mm, anther ca. 1 mm long. Ovary ca. 2 mm in diam., style ca. 9–10 mm long, glabrous. Mericarps dark puberulous, middle wing obovate, puberulous. apex rounded, margin repand, 1.7–2 × 1–1.2 cm, lateral wings oblong, apex margin crest, 0.9–1.1 × 0.5–0.7 cm.

**Figure 2.**
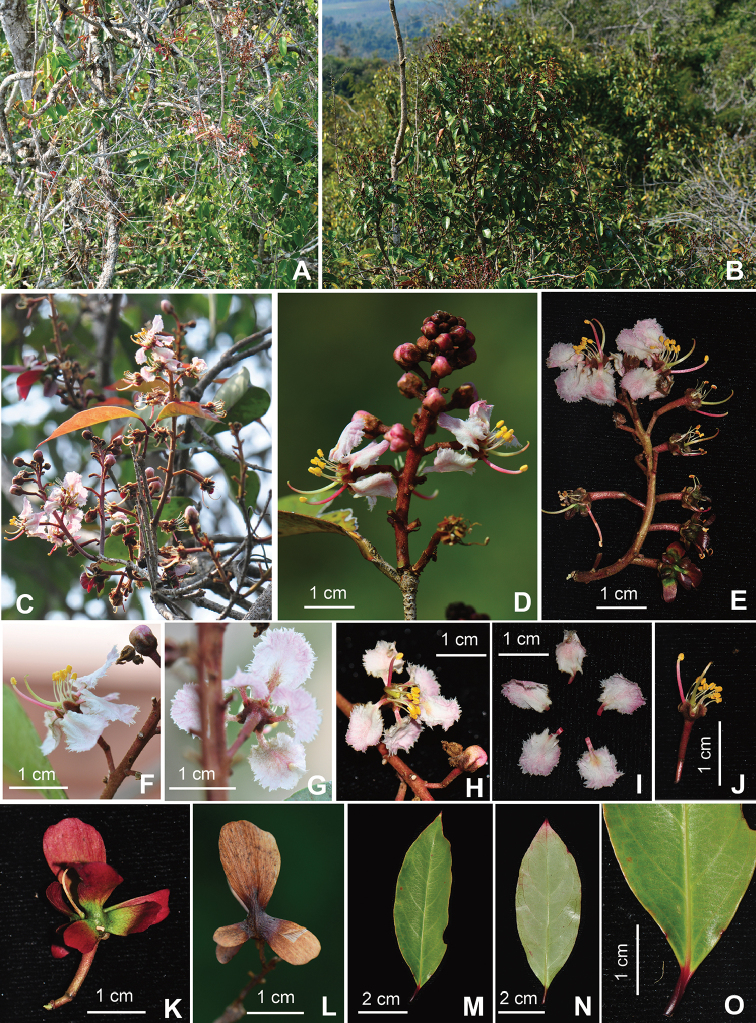
*Hiptageferruginea* Y.H. Tan & Bin Yang, sp. nov. **A, B** Habit **C** Flowering branch **D, E** Inflorescence **F** Flower (lateral view) **G** Flower (dorsal view) **H** Flower (frontal view) **I** Petals **J** Flower (picked petals) **K, L** Samaras **M** Leaf (abaxial view) **N** Leaf (adaxial view) **O** Leaf base (showing the marginal glands). Photographed by B. Yang.

#### Phenology.

Flowering in March and fruiting occurs from March to April.

#### Etymology.

The specific epithet is derived from its inflorescence rust-coloured hairy, ferruginous appearance.

#### Distribution and habitat.

There is only one population and less than 10 individuals known from the limestone areas in the Menglun Natural Reserve.

#### Conservation status.

Since we have not discovered the wild population of this species in other places, very few details about its natural distribution and population status are currently obtained and the information is too inadequate to assess its risk of extinction. At present, it is proposed that it be considered as a taxon under the Data Deficient (DD) category on the basis of current IUCN Red List Categories and Criteria (IUCN 2012).

##### Key to the species of *Hiptagepauciflora*, *H.ferruginea* and their closely related species

**Table:** 

1	Calyx glands conspicuous	**2**
–	Calyx glands inconspicuous or absent	**4**
2	Calyx glands more or less decurrent on the pedicel, middle wings of samara 3–5 cm long	*** H. benghalensis ***
–	Calyx glands scarcely decurrent to pedicel, middle wings of samara less than 3 cm long	**3**
3	Leaf blade ovate or ovate-elliptic to elliptic-oblong, less than 10 cm long, bracteoles lanceolate, 7–11 mm, leaf basal gland dots absent, inflorescence with fewer flowers, usually 1–8 flowers, pedicels 1.8–2.9 cm	*** H. pauciflora ***
–	Leaf blade oblong, 12–13 cm long, bracteoles small, ca. 1mm, leaf basal gland dots 1 pair, inflorescence with many flowers, pedicels ca.1 cm	*** H. multiflora ***
4	Leaf marginal gland dots absence and with 1–2 pairs larminal gland dots	*** H. calcicola ***
–	Leaf marginal gland dots conspicuous, usually 1 pair and larminal gland dots absent	*** H. ferruginea ***

## Discussion

The zygomorphic or mirror-image flower of the *Hiptage* is a unique feature of evolutionary and biological importance that enhances adaptations (Ren et al. 2013, Qian et al. 2016). The presence or absence of calycinal glands calyx is an important characteristic and feature of the glands’ shape and number, and whether it is adnate to pedicel or not is also a key diagnostic for species identification (Sirirugsa 1991, Srivastava 1992, Chen and Funston 2008). Morphologically, *H.pauciflora* shares certain characteristics with *H.benghalensis* in having elliptic-oblong leaf blades and sub-orbicular petals and also shares similarities with *H.multiflora* in having leaf marginal gland dots, sub-orbicular calyx glands and not decurrent to pedicel, obovate middle wing of samara. *Hiptageferruginea* is similar to *H.calcicola* in elliptic leaf shape, hairy pedicels and hairy calyxes without glands, suborbicular petals. The detailed comparisons of the morphological differences amongst these taxa are given in Table 1 and evidence from morphological analysis supports the recognitions of *H.pauciflora* and *H.ferruginea* as two distinct species, respectively.

**Table 1.** Morphological comparison of key characters in *Hiptagepauciflora*, *H.ferruginea* and the morphological similar taxa.

Character	* Hiptage pauciflora *	* H. benghalensis *	* H. multiflora *	* H. ferruginea *	* Hiptage calcicola *
Leaf blade	ovate or ovate-elliptic to elliptic-oblong, 6.0–10.5 × 2.5–12.0 cm	oblong, elliptic-oblong, or ovate-lanceolate, 9–18 × 3–7 cm	oblong, 12–13 × 5–5.5 cm	elliptic or elliptic-oblong, 6.0–7.5 × 2.2–2.8 cm	elliptic or ovate, 3–10.5 × 1–4.5 cm
Leaf marginal gland dots	3–6 pairs	2–8 pairs	2–4 pairs	1 pair	absent
Leaf basal gland dots	absent	1 pair	1 pair	absent	absent
Larminal gland dots	absent	absent	absent	absent	1–2 pairs
Bracteoles	lanceolate, 7–11 × 1–3 mm	acute, ca. 1mm	ovate-triangular, ca.1 mm	ovate-triangular, ca.1 mm	acute, ca. 1mm
Flowers	pink	white	white?	pink to pinkish-white	white
Pedicels	pubescent,1.8–2.9 cm	pubescent, 0.8–2.5 cm	puberulent, ca. 1 cm	dark red hairy, 0.5–1.3 cm	pubescent, 0.4–2 cm
Calyx glands	ovate or sub-orbicular to cordate, scarcely decurrent to pedicel	elliptic, oblong, triangular, lanceolate to oblanceolate; more or less decurrent on the pedicel	sub-orbicular; not decurrent to pedicel	absent	inconspicuous or absent
Sepals	elliptic to ovate-elliptic	broadly elliptic or ovate	ovate	elliptic to ovate-elliptic	elliptic
Petals	ovate-oblong to sub-orbicular	ovate-oblong to suborbicular, glabrous	unknown	suborbicular, 7–8 × 6–7 mm	suborbicular, 3.5–4 × 3.5–4 mm
Claw	1–1.5 mm	1–2 mm, glabrous	unknown	2.5–4 mm, pubescent	1–2 mm, hairy
Middle wings of Samara	obovate-elliptic, 1.7–2.5 × 1–1.3 cm	oblong, elliptic or obovate-lanceolate, 3–5 × 1–1.6 cm	obovate, 2.2–2.5 × 0.7–1.0 cm	obovate, 1.7–2 × 1–1.2 cm	oblong, 2–2.5 × 0.5–0.8 cm

## Supplementary Material

XML Treatment for Hiptage pauciflora

XML Treatment for Hiptage ferruginea
